# From Lab to Clinic: Success Stories of Repurposed Drugs in Treating Major Diseases

**DOI:** 10.1155/adpp/1070716

**Published:** 2025-10-19

**Authors:** Md Sadique Hussain, Prince Ahad Mir, Nishant Kumar, Roohi Mohi-ud-din, Adil Farooq Wali, Reyaz Hassan Mir, Sirajunisa Talath, Sathvik B. Sridhar, Javedh Shareef, Manjunatha Goud, Imran Rangraze, Mohamed El-Tanani, Der Jiun Ooi

**Affiliations:** ^1^ Uttaranchal Institute of Pharmaceutical Sciences, Uttaranchal University, Dehradun, Uttarakhand, 248007, India, uttaranchaluniversity.ac.in; ^2^ School of Pharmaceutical Sciences, Lovely Professional University, Phagwara, 144411, Punjab, India, lpu.in; ^3^ Khalsa College of Pharmacy, G.T. Road, Amritsar, Punjab, 143001, India; ^4^ Department of General Medicine, Sher-I-Kashmir Institute of Medical Sciences (SKIMS), Srinagar, Jammu and Kashmir, 190001, India, skims.ac.in; ^5^ Department of Pharmaceutical Chemistry, RAK College of Pharmacy, RAK Medical and Health Science University, Ras Al Khaimah, UAE; ^6^ Pharmaceutical Chemistry Division, Department of Pharmaceutical Sciences, University of Kashmir, Hazratbal, Srinagar, Jammu and Kashmir, 190006, India, kashmiruniversity.net; ^7^ Department of Clinical Pharmacy & Pharmacology, RAK College of Pharmacy, RAK Medical & Health Sciences University, Ras Al Khaimah, UAE, rakmhsu.com; ^8^ RAK College of Medical Sciences, RAK Medical and Health Sciences University, Ras Al-Khaimah, UAE; ^9^ RAK College of Pharmacy, RAK Medical & Health Sciences University, Ras Al Khaimah, UAE, rakmhsu.com

**Keywords:** computational modelling, drug development, drug repurposing, high-throughput screening, personalized medicine, therapeutic indications

## Abstract

Drug repurposing, the process of identifying new therapeutic uses for existing drugs, has emerged as a cost‐effective and time‐saving alternative to traditional drug development. This strategy leverages the known pharmacological and safety profiles of approved or investigational drugs to accelerate their clinical application for other diseases. In recent years, repurposed drugs have played a crucial role in addressing treatment gaps in complex and multifactorial diseases such as cancer, neurodegenerative disorders and infectious diseases. This review highlights prominent examples where repurposed drugs have successfully transitioned from laboratory findings to clinical application. We discuss key molecular mechanisms, including polypharmacology and target pathway modulation that enable repositioning. Emphasis is also placed on advances in computational approaches, network pharmacology and data‐driven tools that enhance repurposing efforts. Additionally, we outline the challenges related to regulatory hurdles, intellectual property and clinical validation. By analysing these success stories, we aim to provide a strategic framework to guide future drug repurposing initiatives.

## 1. Introduction

Since the onset of the COVID‐19 pandemic, drug repurposing (DRP) has received growing attention as a complementary strategy to novel drug discovery for identifying potential treatments and supportive therapies. Repurposing existing drugs, already approved by regulatory bodies for different indications, offers a promising and pragmatic approach that bypasses several early‐stage development hurdles [1]. The key advantages of DRP include reduced development time and costs, improved safety assurance and a higher probability of regulatory approval compared to de novo discovery [2–4]. These advantages stem from the availability of previously approved dosing and safety data, as well as insights obtained from preclinical toxicology and clinical trials (Phase I–III) for the drug’s original indication. Despite advances in molecular biology, bioinformatics and therapeutic target identification, the translation of scientific discoveries into effective therapies remains challenging [5–7]. The pharmaceutical industry continues to face obstacles such as high attrition rates, lengthy development timelines in complex disease areas and evolving regulatory landscapes, all of which contributing to increasing R&D expenditures [8, 9]. In many cases, the return on investment in drug development has been reported to be lower than the capital invested, discouraging innovation and reducing market competitiveness [10].

Against this backdrop, DRP has emerged as a cost‐efficient alternative that maximizes the therapeutic potential of known molecules [5]. It is particularly useful in rare diseases and emerging infectious diseases, where traditional drug development pipelines may be impractical [2]. The rapid deployment of repurposed agents during the COVID‐19 pandemic, such as remdesivir, tocilizumab and dexamethasone, highlighted the clinical and economic value of this approach. These examples underscore how DRP can address urgent health needs when time and safety assurance are critical [2, 11–13].

**Figure 1:**
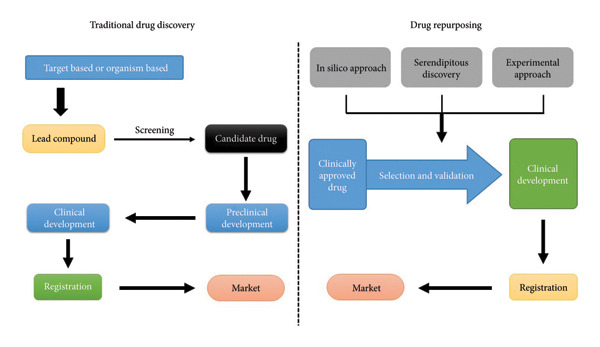
Traditional drug discovery develops new drugs; drug repurposing finds new uses for existing drugs.

Furthermore, DRP is supported by a growing arsenal of advanced technologies. Computational tools, systems pharmacology, omics integration and machine learning (ML) are now central to identifying viable candidates for repositioning. These approaches enable target prediction, mechanism‐of‐action elucidation and phenotypic screening with enhanced accuracy. Complementing in silico methods, high‐throughput screening and data mining of clinical trial databases offer additional avenues for validating repurposing hypotheses [14]. While regulatory and phase III costs may remain similar to those of new drugs, substantial savings can be achieved in preclinical and phase I and II expenses. Collectively, these advantages can lead to a less risky and more expedited return on investment in repurposed drug development, with lower average costs once failures are factored in. Moreover, repurposed drugs may uncover new therapeutic targets and pathways for further exploration and exploitation in drug development. Figure 1 shows the difference between traditional drug discovery and DRP.

Despite these advancements, DRP still faces notable challenges. Intellectual property protection, limited commercial incentives, regulatory ambiguities and a lack of standardized evaluation frameworks may hinder successful translation. However, ongoing policy reforms, collaborative academic‐industry models and data‐sharing initiatives are working to overcome these barriers and foster innovation. Nevertheless, despite the promising pipeline of novel drug candidates, the failure rate in late‐stage clinical trials continues to hinder therapeutic advancement. Consequently, there is a growing imperative to explore innovative and efficient drug development strategies. Drug repurposing offers a pragmatic and strategic solution by mitigating the high costs, long timelines and risks associated with traditional drug discovery. This approach is especially relevant in addressing urgent healthcare challenges such as antimicrobial resistance, rare diseases and rapidly emerging viral outbreaks, where swift therapeutic responses are critical. The motivation for this review stems from the need to systematically evaluate successful DRP efforts that have already transitioned from bench to bedside, intending to extract translational insights that can inform future innovation.

### 1.1. Historical Context and Evolution of Drug Repurposing

Throughout history, DRP has often occurred opportunistically and by chance. When a drug exhibited an unintended off‐target effect or a newly discovered on‐target effect, it was then explored for potential commercial use. Notably, many successful instances of DRP were not the result of a systematic approach. For instance, sildenafil citrate, initially developed as an antihypertensive medication, found unexpected success in treating erectile dysfunction after retrospective clinical observations. Similarly, thalidomide, originally introduced as a sedative but later withdrawn due to its association with severe birth defects, was fortuitously repurposed for conditions such as erythema nodosum leprosum (ENL) and multiple myeloma (MM) [5]. Thalidomide, originally withdrawn due to teratogenic effects, received FDA approval in 1998 for ENL and in 2006 for MM, following clinical trials demonstrating significant improvements in progression‐free survival (PFS) [15].

Sildenafil, marketed as Viagra for erectile dysfunction treatment by Pfizer, captured a significant market share, generating worldwide sales of $2.05 billion in 2012 [16]. Thalidomide, despite its initial setbacks, emerged as an effective therapy for ENL in 1964 and later for MM in 1999 [5, 17]. Its success led to the development of derivative drugs like lenalidomide (Revlimid, Celgene), which achieved global sales of $8.2 billion in 2017 [18]. While some successful DRP cases stemmed from an understanding of drug pharmacology or retrospective clinical analyses, others resulted from more systematic approaches. These systematic approaches have identified numerous promising candidate drugs, many of which are undergoing advanced clinical trials for various diseases, both common and rare. However, significant technical, regulatory and organizational challenges persist, hindering the progress of DRP efforts.

## 2. Methodology

This narrative review was designed to highlight clinically validated and translationally significant examples of DRP across a range of major diseases. To ensure relevance and rigour, we selected case studies based on the following general criteria: (1) evidence of regulatory approval or clinical trial progression for a new indication; (2) support from peer‐reviewed publications, clinical databases (e.g., ClinicalTrials.gov) or drug regulatory authorities (e.g., FDA and EMA) and (3) therapeutic significance in addressing high‐burden diseases such as cancer, metabolic disorders, neurodegenerative conditions and infectious diseases. Literature was identified using nonsystematic searches of PubMed, Scopus, Google Scholar and official regulatory agency databases. The timeframe considered was primarily from 1990 to 2024, with emphasis on well‐documented repurposing success stories. Priority was given to drugs supported by mechanistic insights, clinical trial data or real‐world effectiveness. Studies in English and human subjects were preferred. While not exhaustive, the selection reflects examples that collectively demonstrate the clinical, regulatory and strategic dimensions of modern DRP efforts.

## 3. Mechanisms and Rationale of Drug Repurposing

The journey of drug discovery and development is intricate and protracted, spanning approximately 10–15 years and demanding an investment exceeding $1 billion [19, 20]. It commences with the identification of a target, which could be a molecule or pathway implicated in a disease process. Subsequently, lead compounds are identified, representing molecules that interact with the target and hold the promise of becoming therapeutic agents. These lead compounds undergo refinement through hit‐to‐lead optimization, a process involving molecular modifications aimed at enhancing their properties and suitability for drug use [21]. Following the identification of a lead compound, it undergoes preclinical evaluation in laboratory settings to assess its safety and efficacy. This entails testing the compound in cell cultures, animal models and in vitro systems to ascertain its pharmacokinetics, pharmacodynamics and toxicity profile [22]. The outcomes of these assessments inform decisions regarding the compound’s advancement to clinical trials.

Clinical trials proceed through three sequential phases. Phase I trials entail testing the compound in a small cohort of healthy volunteers to assess its safety, dosage parameters and side effects. Phase II trials extend the evaluation to a larger group of patients affected by the disease to gauge both efficacy and safety within real‐world contexts. Phase III trials further validate safety and efficacy in a substantial patient population while also gathering long‐term safety and effectiveness data [23]. Upon completion of clinical trials and approval by regulatory bodies, the drug can be manufactured and brought to market. Despite the complexity and resource‐intensive nature of the drug development process, the potential benefits are profound, with new drugs holding the potential to significantly impact public health and enhance quality of life [19, 20, 24].

In recent years, there has been notable attention directed toward this approach, especially in the treatment of cancer, cardiovascular diseases (CVDs), neurodegenerative disorders (NDs) and various infectious diseases such as COVID‐19 [25]. The rationale behind DRP stems from comprehending the pathophysiological mechanisms of diseases and pinpointing potential therapeutic targets within these mechanisms. The effectiveness of DRP hinges on the wealth of information available regarding the beneficial properties, adverse effects and pharmacological characteristics of repurposed drugs. This abundance of data enhances the likelihood of approval in clinical trial phases by furnishing a robust basis for assessing the potential efficacy and safety of the repurposed drug. Recent progress in DRP is grounded in patient‐centric methodologies, underpinned by systematic, translational drug targeting practices [26].

### 3.1. Molecular Mechanisms Underlying Drug Repurposing

**Figure 2:**
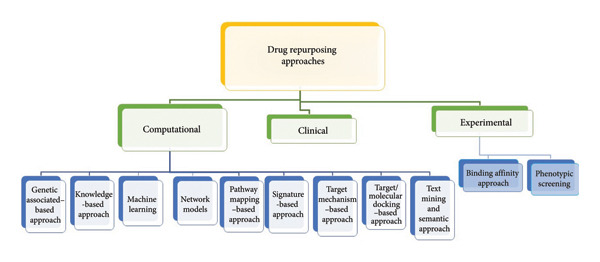
Methods for drug repurposing include computational approaches, biological assays and clinical observations.

The process of DRP hinges on uncovering existing drugs suitable for novel indications, often by investigating their interactions with target proteins, pathways or diseases. Figure 2 shows the different approaches for the DRP. CTs, such as molecular docking and molecular dynamics simulations, play a pivotal role in predicting the potential of drugs to bind to specific targets and comprehending their molecular‐level interactions. In the realm of cancer treatment, the concept of DRP has surfaced as an auspicious strategy owing to the constraints associated with conventional cancer treatment modalities such as surgical excision, chemotherapy and radiotherapy [27]. The process of developing a novel drug for cancer therapy is protracted, financially burdensome and inherently inefficient. In contrast, DRP presents a swifter and more economical avenue for discovering novel antineoplastic applications for pre‐existing drugs, thereby aiding in surmounting therapy resistance [28, 29]. CTs have been instrumental in deciphering cancer biology and pinpointing potential repurposed drugs. For instance, niclosamide has emerged as a promising candidate for treating NDs like Parkinson’s disease (PD) and amyotrophic lateral sclerosis [30]. In the domain of NDs such as schizophrenia, the concept of DRP holds promise in expediting clinical trials and mitigating the substantial financial risk associated with failed drug discovery endeavours. Within the realm of schizophrenia drug discovery, uncovering fresh therapeutic applications for sanctioned drugs has proven instrumental in tackling elevated levels of treatment resistance and refractory symptoms [31]. DRP has been a subject of exploration for conditions such as traumatic brain injury. Drugs like edaravone, glyburide, ceftriaxone, levetiracetam and progesterone have been scrutinized for their prospective neuroprotective properties [32]. In addressing infectious diseases, DRP strategies have been applied to SARS‐CoV‐2 and its NDs. Studies have identified potential candidates like carvedilol, andrographolide, 2‐methoxyestradiol, etanercept, polaprezinc and arsenic trioxide [33]. In the context of COVID‐19, there has been an exploration of DRP strategies aimed at identifying broad‐spectrum antivirals. These strategies encompass the utilization of both direct‐acting repurposed antivirals (DARA) and host‐targeting repurposed antivirals (HTRA). The impetus behind this approach stems from the sluggish pace observed in the discovery of novel antiviral agents, coupled with the high rates of disuse and significant costs linked with developing new antiviral medications [34]. Regarding autoimmune diseases, DRP endeavours have targeted conditions like type 2 diabetes. Food and Drug Administration (FDA)–approved drugs have been investigated for their ability to inhibit α‐glucosidase, an enzyme crucial in carbohydrate degradation [35]. In essence, DRP involves repurposing existing drugs for new indications by exploring their interactions with target proteins, pathways or diseases. CTs facilitate this process, which spans across various disease domains, encompassing cancer, NDs, infectious diseases and autoimmune diseases.

Molecular docking simulations estimate the spatial fit and binding energy between repurposed drugs and new targets, often serving as a preliminary screening tool. MD simulations further evaluate the stability of drug–target interactions over time, providing insights into binding kinetics and conformational flexibility. These simulations have been particularly instrumental in oncology, where drug candidates are screened against oncogenic kinases and transcription factors. For example, studies have shown that niclosamide exhibits high binding affinity and sustained interaction with Wnt/β‐catenin pathway proteins in colorectal cancer (CRC) models [36], as validated through MD trajectories and hydrogen bond occupancy analysis.

## 4. Approaches and Strategies for Identifying Repurposed Drugs

DRP involves identifying new therapeutic applications for drugs that have already received approval for other uses. This approach appears to streamline the drug discovery process by accelerating it and bypassing the need for preclinical and clinical studies related to ADMET properties and drug optimization challenges, thereby improving the success rate of drug development [37]. Additionally, recent evidence also indicates a high success rate associated with the DRP strategy [38, 39].

### 4.1. Computational Methods and Bioinformatics Tools

CTs for DRP encompass a variety of methods such as molecular docking, ML and in silico approaches. These methodologies are utilized to screen approved drugs or potential candidates for their antiviral properties, which can then be repurposed to address emerging diseases like COVID‐19. This repurposing process is particularly advantageous in combating infectious diseases as it enables the rapid identification of effective antiviral agents without the necessity for extensive preclinical trials [40]. Molecular docking serves as a method to investigate the binding affinity of a drug molecule with a specific target protein, such as the SARS‐CoV‐2 Main Protease. Utilizing software like AutoDock VINA, this technique predicts the strength of interaction between the drug and the target protein. For instance, in the case of the SARS‐CoV‐2 Main Protease, three drugs (danoprevir, remdesivir and saridegib) exhibited stronger binding affinity than the active Mpro inhibitor, with danoprevir and remdesivir demonstrating the highest binding affinity [41]. ML methods can be applied to identify analogous drugs capable of treating comparable diseases. This entails developing a model to forecast potential candidate drugs for rare orphan diseases by scrutinizing interactions between drugs and various factors like drug–drug, drug–genes, drug–enzymes and drug–targets [42]. In silico strategies, such as structure‐based DRP, offer a means to pinpoint potential drug candidates targeting viral elements like the SARS‐CoV‐2 Main Protease [43]. Employing HTS enables the targeting of essential viral proteins, unveiling potential drug candidates [44]. Advanced AI technologies like ChatGPT can aid in prioritizing DRP candidates by synthesizing scientific insights garnered from extensive internet‐based research. This facilitates the identification of promising drugs for repurposing in diseases such as Alzheimer’s disease (AD) [45, 46]. CTs for DRP have demonstrated efficacy in treating diverse diseases, including cancer, and hold the potential to revolutionize the landscape of drug discovery [47].

Bioinformatics tools play a pivotal role in identifying repurposed drugs for a spectrum of diseases and conditions, spanning from cancer and NDs to COVID‐19. These tools aid in forecasting the bioactivity of compounds derived from natural products and pinpointing potential drug targets. Below are various examples of bioinformatics tools and methodologies employed in DRP. Molecular docking forecasts the binding affinity of a small molecule to a target protein, facilitating the identification of potential drug candidates. It proves particularly advantageous in DRP by enabling the evaluation of existing drugs against novel targets [48]. Computer‐aided drug design (CADD) encompasses CTs like molecular modelling and ML to predict the efficacy of small molecules against specific targets. Widely applied in DRP, CADD assists in discovering new applications for established drugs [49]. RNA‐sequencing data analysis involves scrutinizing gene expression in cells afflicted by particular pathogens, such as SARS‐CoV‐2. By discerning differentially expressed genes and enriched pathways, researchers can forecast drugs that target human proteins within these pathways [50]. Gene Ontology (GO) and Kyoto Encyclopedia of Genes and Genomes (KEGG) analyses serve to unveil biological pathways and gene functions linked with particular diseases or conditions. Through pathway analysis, researchers can pinpoint potential drug targets and repurpose existing drugs for fresh indications [51]. Protein–protein interaction (PPI) network analysis entails scrutinizing interactions among proteins in a cellular network to pinpoint potential drug targets. By delving into these interactions, researchers can unearth hub proteins pivotal to the network, which might serve as suitable targets for DRP [52]. Biomarker‐guided therapies involve identifying biomarkers correlated with specific diseases or conditions and utilizing them to categorize patients into subgroups responsive to repurposed drugs. This enables the development of tailored therapies for NDs and other conditions [53]. These examples merely scratch the surface of the diverse bioinformatics tools and methodologies harnessed in DRP. Leveraging these tools empowers researchers to unearth fresh applications for existing drugs, expedite therapy development and ultimately enhance patient outcomes.

In addition to simple docking scores, modern bioinformatics workflows incorporate transcriptome‐wide differential gene expression analysis and integrate it with protein–protein interaction networks. This allows for the identification of hub genes that may be more relevant to disease phenotypes. Tools like Connectivity Map (CMap) and LINCS L1000 can predict drugs that reverse disease‐specific expression profiles [54]. When combined with network pharmacology, such predictions form the basis for prioritizing repurposing candidates. For instance, transcriptomic reversal scoring has identified topiramate as a candidate for inflammatory bowel disease [55], with supporting evidence from downstream cytokine profiling.

### 4.2. High‐Throughput Screening and Phenotypic Assays

HTS plays a pivotal role in DRP strategies by enabling the swift identification of promising therapeutic candidates from existing drugs or drug‐like substances. This methodology proves especially beneficial in combating infectious diseases, where the urgent demand for novel treatments calls for the repurposing of existing drugs. In the realm of cancer research, HTS has facilitated the discovery of selective inhibitors targeting exosome biogenesis and secretion, presenting potential for advanced cancer therapy [56]. Similarly, in NDs, HTS methodologies have been instrumental in scrutinizing drug libraries to pinpoint compounds capable of disrupting interactions between 14‐3‐3 protein and BAD, offering prospects for treatment [57]. Addressing infectious diseases such as COVID‐19, HTS techniques have been employed to sift through drug libraries for compounds capable of inhibiting the protease activity of TMPRSS2, a crucial player in SARS‐CoV‐2 entry [58]. Additionally, HTS has identified compounds capable of inducing cancer cell death, thus holding promise for CRC treatment. In the domain of autoimmune diseases, HTS has been pivotal in screening drug libraries for compounds that interfere with 14‐3‐3 protein: BAD interactions, potentially serving in CRC treatment [57]. Nonetheless, further research is warranted to pinpoint specific drugs or drug‐like compounds suitable for repurposing in autoimmune diseases. HTS stands as a valuable asset in DRP endeavours, particularly concerning infectious diseases, cancer, NDs and autoimmune diseases. By systematically analysing existing drugs or drug‐like substances, researchers can swiftly unearth potential therapeutic candidates amenable to repurposing for novel indications.

Phenotype‐based screening proves advantageous in situations where there is limited or absent information regarding the target. Essentially, this method entails the random screening of extensive compound libraries [59]. However, this approach may obscure the molecular mechanism of drug action and the target involved. Compounds identified through this method have the potential to interact with multiple proteins and diverse pathways [60]. Numerous instances exist where drugs have been discovered through this avenue, with aspirin serving as a prime example, as its mode of action was elucidated nearly a century post its approval [61, 62]. A key advantage of this strategy lies in its applicability across various diseases, circumventing the need for prior knowledge about their specific targets.

### 4.3. Drug Repositioning in Clinical Trials

DRP in clinical trials involves identifying new actionable targets for specific diseases and developing inhibitors to resensitize drug‐resistant tumours or induce synthetic lethality in tumours with particular oncogenic mutations. In the realm of cancer, advancements in HTS have notably enhanced therapy, particularly in a personalized approach, targeting defined patient subgroups with specific mutations [63]. NDs are increasingly understood through the concept of Aberrant Cell Cycle Diseases (ACCD), revealing a shared mechanism of aberrant cell cycle re‐entry characterized by kinase/oncogene activation and tumour suppressor inactivation. Leveraging cancer therapies like kinase inhibition and tumour suppressor elevation holds promise for NDs [64]. While the US FDA has greenlit 74 kinase inhibitors, the shortage of FDA‐approved drugs for NDs like AD underscores significant unmet needs [65]. In infectious diseases, swift clinical trials have been launched to assess repurposed drugs with plausible biological effectiveness against emerging viruses like COVID‐19. Ranging from small single‐site studies to large global randomized trials, these endeavours provide reliable estimates of efficacy. Simultaneously, collaborations between government and industry have accelerated the development and evaluation of vaccines to mitigate the risk of severe illness [66]. In autoimmune diseases, the advent of induced Pluripotent Stem Cells (iPSCs) technology allows for patient‐specific disease models in drug screening and development. This technological progress extends to NDs such as AD, PD, ALS and FRAX, offering tailored approaches to therapy development [67]. DRP in clinical trials represents a promising avenue for novel therapies across various diseases. HTS, including DRP, holds potential for significant therapeutic advancements, especially within the realm of personalized medicine (PM) and the discovery of new therapeutic targets.

One noteworthy instance of drug repositioning in clinical trials is the repurposing of antiviral drugs for combating COVID‐19. Researchers have pinpointed cellular pathways exploited by SARS‐CoV‐2, the virus responsible for COVID‐19, which coincide with pathways implicated in cancer cells. Utilizing cancer drugs targeting these pathways, promising clinically available drugs have been swiftly repurposed to address COVID‐19 [68]. Another illustration is the adoption of interferon‐free combination therapy alongside DAARs for hepatitis C virus (HCV) treatment. The INFORM‐1 trial demonstrated that a combination of a nucleoside analogue polymerase inhibitor and a protease inhibitor, without polyethylene glycol (PEG) or ribavirin (RBV), effectively reduced HCV viral load by 5 1 log10 IU/mL in 14 days with no emergence of resistant virus. This therapy has rendered HCV undetectable in most patients, offering a more potent and expedited treatment approach [69]. In the realm of autoimmune diseases, autoantibodies (autoAbs) serve as inclusion criteria in clinical trials, and their presence is essential for prescribing certain new drugs. The accurate interpretation of autoAbs hinges on the detection technique employed and its individual characteristics [70]. Standardization of assays for autoAbs is increasingly imperative to bolster their reliability as biomarkers for systemic rheumatic diseases [71, 72]. Within oncology, the utilization of locked nucleic acid (LNA)–modified antisense oligonucleotides as anticancer agents exemplify drug repositioning. These antisense molecules proficiently bind and inhibit target mRNAs or microRNAs, pivotal for cancer cell malignant transformation or survival. Advances in oligonucleotide chemistry have yielded molecules with enhanced stability and affinity, thus yielding more potent anticancer therapies [73]. Drug repositioning in clinical trials has yielded notable successes in addressing COVID‐19, HCV, autoimmune diseases and cancer. By repurposing existing drugs for new applications, researchers have developed more potent and expeditious treatments for diverse diseases.

## 5. Examples of Successful Drug Repurposing

### 5.1. Cancer Drugs in Nononcological Conditions

By adopting an alternative technique, which is to repurpose cancer drugs for use in disorders that are not connected to oncology, it is feasible to provide a broader number of alternatives for mitigating the effects of cancer (Table 1). The significant breakthrough was achieved while undergoing COVID‐19 therapy. Interleukin inhibitors, like IL‐6 or IL‐6 receptor blocking antibodies (Abs), were used in COVID‐19 therapy [113]. These Abs include tocilizumab (Actemra), sarilumab (Kevzara) and siltuximab (Sylvant), all of which have been reviewed and authorised by the FDA for the treatment of a range of conditions. Castleman’s syndrome, smouldering MM and lymphoproliferative diseases are some of the ailments that fall into this category [74]. Many JAK inhibitors, including ruxolitinib, have shown promise as possible treatments for COVID‐19. For the treatment of primary myelofibrosis and polycytheamia vera, these medications have previously been granted a licence. Steroids and selinexor, a selective nuclear export inhibitor, have been licenced for use in the treatment of MM patients who have had a relapse or who have been resistant to previous treatments [75, 114]. On the other hand, there is a growing body of evidence that shows that XPO‐1 inhibitors may directly reduce the reproduction of some viruses by limiting nuclear transport. These viruses include the human immunodeficiency virus (HIV), influenza, rabies, dengue and cytomegalovirus (CMV) [76]. The immunosuppressive regimens that contain etoposide and methotrexate, which are often used to treat lung and testicular malignancies, are being used to treat cytokine storms in individuals who have been diagnosed with COVID‐19 [77]. The combination of methotrexate (an antimetabolite that kills cancer cells by preventing them from making DNA) and misoprostol represents a safe and effective alternative to invasive methods for the termination of early pregnancy [78].

**Table 1:** Examples of successful drug repurposing.

Class of drugs	Name of the drug	Original indication	After repurposing used as	Reference
Cancer drugs in non‐oncological conditions	Sarilumab siltuximab tocilizumab	Antibodies	Castleman’s syndrome, smouldering MM and lymphoproliferative diseases	[74]
	Ruxolitinib	Antibody	COVID‐19	[75]
	Steroids and selinexor	Selective nuclear export inhibitor	Multiple myeloma (MM)	[75]
	Felezonexor	Nuclear export protein exportin‐1 inhibitor	Reduce the reproduction of some viruses like human (HIV), influenza, rabies, dengue and cytomegalovirus.	[76]
	Etoposide and methotrexate	Immunosuppressive	To treat cytokine storms in individuals who have been diagnosed with COVID‐19	[77]
	Methotrexate and misoprostol	Antimetabolite	Alternative to invasive methods for the termination of early pregnancy	[78]

Antiviral drugs for nonviral diseases	Ribavirin (RBV)	Antiviral	Inhibiting tumour proliferation	
	Interferon‐alpha	Anticancer	Hinder cancer stem cell growth by targeting viral proteins crucial for their regulation	[79, 80].
	Remdesivir	Antiviral	Suppressing the production of proinflammatory cytokines	[81–83]
	Cidofovir	Antiviral	Anticancer properties against ovarian and cervical cancers	[84]
	Oseltamivir	Antiviral	Reducing neuroinflammation severity in mice with multiple sclerosis (MS)	[80, 85]
	Lopinavir	Antiviral	Decrease amyloid‐beta production, a protein linked to AD	[86]
	Acyclovir	Antiviral	Alleviate neuroinflammation and offer neuroprotective benefits in disorders like AD and MS	[87–90]
	Valacyclovir	Antiviral	Enhancing cognitive function in animal models of AD	[90, 91]
	Zanamivir’s	Antiviral	Lowering CVDs risks in chronic kidney disease patients	[92–94]
	Rituximab	Antibody	Mitigate rheumatoid arthritis (RA) severity by targeting disease‐involved B cells.	[95]

Psychiatric medications for neurological disorders	SSRIs and SNRIs	Depression	Potential neuroprotective and neurorestorative effects in conditions such as PD, AD and stroke	[96]
	Fluoxetine	Depression	Enhancing motor function and reducing neuroinflammation in PD models	[97, 98].
	Clozapine, olanzapine and risperidone	Dyskinesia	Neuroprotective and anti‐inflammatory properties, in NDs like PD, Huntington’s disease and traumatic brain injury	[99].
	Lithium	Bipolar disorder	Neuroprotective effects in conditions such as AD, PD and stroke	[100].
	Valproic acid	Mood stabilizer	Managing migraine, epilepsy and NDs due to its neuroprotective and anti‐inflammatory properties	[101–104].
	Methylphenidate	ADHD	Enhancing cognitive function and reducing apathy in conditions such as AD and traumatic brain injury	[105]
	Fluvoxamine	SSRI	Managing COVID‐19	[106]

Cardiovascular medications for metabolic disorders	Metformin	Diabetes	Anticancer	[107]
	Probenecid	Gout	Cytoprotective	[108, 109]
	Colchicine	Gout	Coronary syndrome	[110]
	Dapagliflozin	Diabetes	Heart failure	[111]
	Exenatide	Diabetes	Acute coronary disease	[112]

### 5.2. Antiviral Drugs for Nonviral Diseases

Originally developed to combat viral infections, antiviral drugs have demonstrated potential for repurposing in the treatment of various nonviral conditions, such as cancer, NDs, inflammatory diseases and CVDs. Numerous investigations have explored the prospect of utilizing antiviral medications in cancer therapy. RBV, an antiviral medication prescribed for hepatitis C, has demonstrated encouraging outcomes in inhibiting tumour proliferation and improving the efficacy of chemotherapy drugs across diverse cancer types, including breast cancer, CRC and leukeamia [79, 115]. For instance, Jamir et al. proposed repurposing antiviral drugs like RBV and interferon‐alpha to target cancer stem cells. These drugs were found to hinder cancer stem cell growth by targeting viral proteins crucial for their regulation [79, 80]. Similarly, Hahn et al. investigated the role of antiviral drugs in augmenting cancer immunotherapy. It revealed that remdesivir, an antiviral drug, could boost T cell activity in cancer patients by suppressing the production of proinflammatory cytokines [81–83]. Cidofovir, an antiviral drug sanctioned for treating CMV infections, has displayed anticancer properties against several solid tumours, such as ovarian and cervical cancers [84].

In the realm of NDs, antiviral drugs have garnered attention. Antiviral medications exhibit promise in tackling NDs, especially those associated with neuroinflammation and neurodegeneration [116]. Oseltamivir exhibited potential in reducing neuroinflammation severity in mice with multiple sclerosis (MS) [80, 85]. Furthermore, research delved into antiviral drug use for AD treatment. Lopinavir, an antiviral drug, was found to decrease amyloid‐beta production, a protein linked to AD [86]. Acyclovir, a commonly prescribed antiviral medication for treating herpes simplex virus (HSV) infections, has been investigated for its potential to alleviate neuroinflammation and offer neuroprotective benefits in disorders like AD and MS [87–90]. Another antiviral drug, valacyclovir, utilized for HSV infections, has demonstrated encouraging outcomes in diminishing neuroinflammation and enhancing cognitive function in animal models of AD [90, 91].

Exploring CVDs, research revealed zanamivir’s potential in lowering CVDs risk in chronic kidney disease patients [92–94]. Moreover, in autoimmune diseases, antiviral drugs like rituximab were investigated. Lopez‐Olivo et al. showed that rituximab could mitigate rheumatoid arthritis (RA) severity by targeting disease‐involved B cells [95]. The reutilization of antiviral medications for nonviral ailments presents numerous benefits, such as decreased developmental expenses, expedited clinical testing durations and enhanced comprehension of the safety profiles of these medications [117, 118]. Nonetheless, it is imperative to undertake comprehensive preclinical and clinical investigations to assess the effectiveness, safety and suitable dosage for these repurposed uses. The effectiveness of DRP initiatives hinges upon a thorough comprehension of the molecular mechanisms and biological pathways implicated in both the initial and proposed therapeutic applications, along with meticulous scientific scrutiny and regulatory endorsements.

### 5.3. Psychiatric Medications for Neurological Disorders

In recent years, there has been notable interest in repurposing psychiatric medications for the treatment of NDs, owing to their potential to modulate various neurobiological pathways and mechanisms relevant to these conditions. Below are examples of psychiatric medications being investigated for repurposing in NDs.

Selective serotonin reuptake inhibitors (SSRIs) and serotonin‐norepinephrine reuptake inhibitors (SNRIs), traditionally prescribed for depression and anxiety disorders, have been studied for their potential neuroprotective and neurorestorative effects in conditions such as PD, AD and stroke [96]. For instance, fluoxetine (an SSRI) has exhibited promise in enhancing motor function and reducing neuroinflammation in PD models [97, 98]. Moreover, atypical antipsychotics, including clozapine, olanzapine and risperidone, have displayed neuroprotective and antiinflammatory properties, rendering them potential candidates for repurposing in NDs like PD, Huntington’s disease and traumatic brain injury [99]. Clozapine, specifically, has been examined for its potential in alleviating dyskinesia (involuntary movements) associated with long‐term levodopa treatment in PD [100, 119].

Lithium, utilized as a mood stabilizer in bipolar disorder treatment, has shown encouraging results in preclinical studies for its neuroprotective effects and potential applications in conditions such as AD, PD and stroke [120]. Valproic acid, another mood stabilizer, has been explored for its potential in managing migraine, epilepsy and NDs due to its neuroprotective and anti‐inflammatory properties [101–104, 121]. Furthermore, Methylphenidate, primarily prescribed for attention deficit hyperactivity disorder, has been investigated for its potential in enhancing cognitive function and reducing apathy in conditions such as AD and traumatic brain injury [105].

Investigations have centred on repurposing psychiatric drugs, including SSRIs, for their potential therapeutic impact in AD. For example, fluvoxamine, an SSRI, has demonstrated effectiveness in managing COVID‐19, and ongoing research is exploring its potential advantages in AD [106]. Furthermore, a study examining the repurposing of the anti‐MS medication fingolimod (FTY720) for AD underscored its potential in addressing inflammatory processes associated with the condition [122]. Investigation has delved into repurposing psychiatric drugs for PD. For instance, a study examining the historical background and potential treatments for PD outlined approaches for DRP, such as utilizing current medications to address particular pathways implicated in the condition [123]. Another study concentrated on DRP for PD through Mendelian randomization of the druggable genome, uncovering potential drug targets [124]. Investigation has concentrated on repurposing psychiatric drugs for their potential therapeutic benefits in epilepsy. For example, a study employing an in silico DRP pipeline for epilepsy amalgamated deep learning (DL) and structure‐based methodologies to pinpoint candidate inhibitors against 24 target proteins associated with epileptogenesis [125]. Additionally, a review elucidating the mechanism of antiseizure medications and emerging patterns in epilepsy management underscored the significance of comprehending the mechanisms underlying these drugs and their prospective utility in epilepsy therapy [126]. MS is an autoimmune condition that impacts the central nervous system. Investigations have delved into repurposing psychiatric drugs for their potential therapeutic benefits in MS. For instance, a study examining the repurposing of the anti‐MS medication fingolimod (FTY720) for AD also examined its potential therapeutic uses in MS [122].

Although DRP holds promise as a strategy for crafting novel treatments for NDs, it is not without hurdles. These obstacles encompass the necessity for a comprehensive grasp of the mechanisms underpinning the medications being repurposed, the risk of off‐target effects and the imperative for rigorous examination and validation of the repurposed drugs. Notwithstanding these challenges, the potential advantages of DRP in formulating fresh treatments for NDs render it a captivating realm of investigation with substantial prospects for progress.

The repurposing of psychiatric medications for NDs is founded on the idea that these drugs can modulate various neurobiological pathways, including neurotransmitter systems, neuroinflammation, oxidative stress and cell survival mechanisms, which are often dysregulated in NDs. However, it is essential to recognize that although preclinical and initial clinical studies have yielded promising outcomes, further rigorous research is imperative to establish the efficacy, safety and optimal dosing protocols for the repurposed use of these psychiatric medications in NDs. Thoughtful consideration of potential side effects and drug interactions is also crucial.

### 5.4. Cardiovascular Medications for Metabolic Disorders

The findings of several studies that were conducted with the intention of locating older drugs for a variety of CVDs have returned equivocal results. Several antidiabetic medications, including metformin or empagliflozin, methotrexate and Abs against TNF‐α or IL‐1β, were tested in these clinical studies. Several of these studies, such as EMPEROR on the effectiveness of empagliflozin, which produced favourable results, gave evidence that DRP is a practical strategy for treatment development in CVDs. This evidence was supplied by the fact that the outcomes of these trials were positive [127]. Metformin, approved by the FDA in 1994 for type 2 diabetes, has shown anticancer potential in multiple observational studies and ongoing clinical trials (e.g., NCT01941953) that suggest reduced cancer incidence and improved overall survival in diabetic patients [107].

Over the course of many decades, probenecid has been the medicine of choice for gout patients who are hoping to prevent attacks. It does this by stopping the kidneys from reabsorbing uric acid and by inhibiting anion transporters, which results in a reduction in the amount of uric acid in the blood. When new drugs such as allopurinol were available, uricosuric agents such as probenecid were proposed as a backup plan for the treatment of gout. In a manner comparable to that of other antigout medications such as canakinumab, colchicine or allopurinol, the repositioning of probenecid for cardiovascular reasons has garnered considerable interest [108, 109]. It has been shown via research that it is capable of exerting its anti‐inflammatory and cytoprotective properties by acting on a wide variety of targets, including purinergic receptors, pannexins and TRPV2 cation channels. Both small‐scale clinical trials and large‐scale cohort studies have shown that probenecid is both safe and effective in treating heart failure (HF) and other CVDs. This has been proven by both types of research procedures. These findings have led us to speculate about the possibility of repurposing probenecid for the treatment of CVDs other than gout and more especially for chronic HF that is brought on by an ischeamic event [128].

Chronic HF with a decreasing ejection fraction is mostly caused by ischaemic heart disease, which is the primary culprit behind this condition. Since survival rates after acute and chronic ischeamic episodes have improved, there has been an increase in the number of cases of HF that are caused by ischeamia. This is a result of developments in therapeutic procedures as well as the use of pharmaceuticals that are more effective in the treatment of heart attacks. Microvascular dysfunction, vasospasm and inflammation have been identified as significant factors in ischeamic heart disease, according to a study that was conducted not too long ago. Over the last several years, the disciplines of medicine development and therapeutic repurposing have shown an increased level of interest in inflammation [129].

It has been shown via preclinical research that inflammatory pathways and mediators play a substantial part in the development of CVDs such as atherosclerosis, myocardial infarction, stroke and HF. Over the course of the last 10 years, there has been a substantial rise in the amount of research that has been conducted with the purpose of examining the possibility of repurposing anti‐inflammatory drugs for the treatment of CVDs. In spite of the optimistic findings from preclinical research, the majority of clinical studies have, up to this point, produced results that are both disappointing and conflicting. The use of TNF‐α inhibitors and the IL‐1β receptor antagonist anakinra demonstrates the need of doing comprehensive research on innovative approaches and inflammatory pathways in the setting of HF [130]. A new approach might be defined as the process of identifying certain patient subgroups that have the potential to benefit from a particular medication. At the primary endpoints, anti‐inflammatory drugs such as canakinumab or colchicine were helpful in lowering the residual inflammatory risk in some categories. This was especially true for those subgroups that had increased levels of highly sensitive C‐reactive protein (CRP) that were higher than the threshold that was specified. On the other hand, these drugs did not seem to lower death rates overall. Because methotrexate did not seem to be effective in the CIRT study, researchers are now looking at the effect that IL‐6 inhibitors, such as tocilizumab and ziltivekimab, have on inflammation that is associated with CVDs [131].

Over the course of human history, the great majority of instances of DRP have been completely unintentional instances. Since epidemiological studies have obtained information on the profiles of pharmacological adverse effects from patients who are already using the medications, this occurs rather often. Several products use this strategy, but one of the most well‐known is sildenafil. The pharmaceutical sildenafil, which was first intended to treat hypertension, has been proven to be equally effective when used to treat erectile dysfunction, according to clinical research. The use of DRP as a strategy has been more prevalent over the course of the last several years. Two methods that are often used include HTS and the computation of drug–target interactions between molecules that are structurally linked to one another [132].

Although CVDs are the leading cause of mortality on a worldwide scale, the pace at which new medications for CVDs are being approved has been steadily decreasing. Although CVDs are the leading cause of death, this continues to be the case. Although CVDs continue to be the leading cause of death, the authors find this to be shocking. As an example, the proportion of new pharmaceuticals that the FDA has approved for the treatment of CVDs has decreased from 16 percent in 1997 to two percent on average in 2018. The situation has deteriorated significantly. According to the accusations that the pharmaceutical sector is now experiencing a “productivity crisis,” the percentage of cancer medications that have been authorised has increased from 16 percent to 27 percent over the course of that time period. Although there has been a significant increase in the number of cancer drugs that have been licenced, this continues to be the case. In an effort to explain this phenomenon, one idea has been proposed that it is made worse by the fact that it is difficult to carry out clinical studies in the cardiology industry. It is common practice for trial regulators to look for “hard” end points rather than biomarker or surrogate outcomes, like levels of CRP or low‐density lipoprotein. Myocardial infarction, stroke and other CVDs that are equivalent are examples of the endpoints that are being discussed here. Because of the rarity of these occurrences in comparison to other end outcomes in domains such as cancer, it is much more critical for studies to have large patient cohorts and extensive follow‐up periods in order to achieve statistical power. When it comes to individuals who are already on conventional medicine, such as statins or ACE inhibitors, this is particularly true. Investigations that involve the CVDs are not only difficult but also expensive since there are a lot of factors that need to be taken into consideration. It is possible that the process of DRP will prove to be a useful method in the area of cardiovascular medicine. This is due to the fact that there is the potential for monetary appreciation [133–135].

Not only is inflammation a significant contributor to the progression of CVDs but it is also one of the most important factors in the formation of CVDs. Inflammation has a significant role in the progression of CVDs. Since it is associated with an increased risk of CVDs, CRP has been the focus of a significant amount of research in the scientific community ever since it was first introduced in 1997. No matter how much lipids are present in the blood or how concentrated they are, this link will continue to exist between the two variables. It has been shown via research that individuals who have a history of inflammatory diseases, such as RA, have a greater likelihood of developing CVDs during the course of their lifetime. This risk is directly proportional to the degree of the inflammatory sickness that is being experienced. Numerous studies indicate that the actual risk is far greater. It has been hypothesised by many studies that the anti‐inflammatory characteristics of statins are a contributing factor to the success of these medications in lowering the risk of CVDs. This is the case although the primary objective of contemporary treatments for CVDs is to reduce the levels of cholesterol in the blood. A pathophysiological process that is referred to as atherogenesis is a main contributor to the overwhelming majority of occurrences of CVDs. These results are supported by the majority of the evidence that was acquired from research that conducted CVD models in animals and in vitro. With the use of these models, we are now able to fully comprehend the significance of inflammatory cells in the process of atherogenesis. Myeloid cells, which include macrophages and monocytes, can transition between M1‐like cells, which are responsible for promoting inflammation, and M2‐like cells, which are responsible for assisting in the repair of tissue and the resolution of inflammation. This is made possible by the phenotypic plasticity that myeloid cells are capable of undergoing. Because of this feature, myeloid cells are able to contribute to the repair of tissues and the resolution of inflammation. These cells become appealing therapeutic targets due to the fact that they have the capacity to function within the context of the pathogenic inflammation that is associated with atherosclerosis. The inflammatory component of CVDs (vascular disease) has been the subject of several therapeutic interventions, with varied degrees of effectiveness, as a result of this. The reduction of the inflammatory component has been the primary focus of these strategies [135–138].

## 6. Challenges and Limitations

### 6.1. Intellectual Property (IP) and Regulatory Issues

DRP, sometimes referred to as drug repositioning, is the process of discovering novel therapeutic applications for established medications that have previously passed safety testing [139]. Repurposed medications have advantages such as decreased research expenses and quicker development schedules in comparison to completely novel treatments. However, they also give rise to certain IP and regulatory concerns [140]. When repurposing a medicine, it is essential to evaluate the current patent landscape to verify that the new use does not violate any existing patents. Occasionally, the expiration of patents for the first application of a medicine may facilitate its repurposing without encountering any infringement concerns [141]. Discovering a new therapeutic application for a medication may allow for obtaining patents, as long as it satisfies the requirements of innovation, nonobviousness and industrial applicability. These patents protect rivals and enable the exclusive sale of the repurposed medicine for the new indication. Patent holders of the original medicine may oppose efforts to repurpose it for new uses, particularly if the repurposed usage infringes on their IP rights (IPR) [142]. Legal disputes over patent infringement or validity may arise, which might possibly impede or delay the development and commercialisation of the repurposed medicine [143]. Regulatory authorities have the authority to provide data exclusivity, in addition to patents, for a certain duration. This means that during this time, rivals are not allowed to use the original company’s data to support their applications for marketing clearance [144]. The exclusivity may impact the timing of market launch for rivals aiming to reuse the same medication. Medications that have been repurposed may qualify for accelerated regulatory procedures, such as the FDA’s 505(b)(2) pathway in the United States or the European Medicines Agency’s (EMA) regulatory framework for repurposed medications [145]. These approaches enable sponsors to partially depend on pre‐existing medication data, which has the potential to expedite the approval process. Although repurposed medications may have the advantage of existing safety data, it is still required to conduct further clinical studies to determine the effectiveness and safety of the drug for the new use [146]. The process of designing and executing these trials involves meticulous deliberation of issues such as patient demographics, outcomes and comparator therapies. Regulatory bodies often mandate the need for modified labelling and packaging to accurately represent the new indication and any relevant safety information. It is crucial to ensure adherence to these rules in order to get marketing permission and conduct postmarket monitoring [147]. Pharmacovigilance programmes are implemented by regulatory bodies to ensure ongoing monitoring of the safety and effectiveness of repurposed medications, even after they have been approved. Sponsors are required to quickly disclose any adverse occurrences or new safety concerns [148].

Before undertaking repurposing initiatives, it is essential to do a thorough freedom to operate (FTO) examination. This study entails evaluating not only current patents but also prospective patent applications, regulatory exclusivities and other IPR that might affect the feasibility of bringing the repurposed medicine to market [143, 149]. Pharmaceutical firms may sometimes have to engage in negotiations with patent holders or other parties that own the necessary IPR to get approval to repurpose a medicine for a different medical use. These agreements sometimes need intricate discussions over issues like as royalties, exclusivity and geographical rights. In addition to patents, pharmaceutical businesses may also depend on trade secrets and private knowledge about medication composition, manufacturing procedures and clinical trial data [150]. Safeguarding these resources is crucial for preserving a competitive edge in the market, especially for repurposed medications where rapid market entry is vital.

Physicians may engage in off‐label usage, which refers to the practice of prescribing medications for purposes that have not been officially authorized by regulatory bodies. Although off‐label usage is permissible and prevalent, pharmaceutical corporations are prohibited from actively endorsing or advertising medications for off‐label use without obtaining regulatory clearance [151]. Effective management of the off‐label use of repurposed pharmaceuticals necessitates the concise transmission of the authorised indications and relevant safety information to healthcare practitioners [152].

### 6.2. Lack of Predictive Models and Biomarkers

The absence of prognostic models in DRP is a substantial obstacle to effectively finding novel therapeutic applications for already pharmaceuticals. The biological systems possess intrinsic complexity, characterised by complicated interplay among genes, proteins, cells and tissues, which have a role in the development of diseases and the response to drugs [153]. In order to effectively replicate illness progression and treatment effects, it is essential for predictive models to include and account for this complexity. Several illnesses have aetiologies that are either poorly understood or complex, which pose challenges in developing reliable prediction models that represent disease processes [154, 155]. Without a thorough comprehension of disease processes, it is difficult to anticipate the interactions between medications and pathogenic pathways, as well as the resulting therapeutic effects. Patients from different populations display notable variations in genetic profiles, environmental influences, lifestyle choices and illness presentations [156]. In order to provide reliable predictions about medication reactions in various patient groups, predictive models need to include and incorporate the differences and variations within these groups. In order to develop predictive models, it is necessary to have access to extensive datasets that include a wide range of biological, clinical and pharmacological information. Nevertheless, these datasets often have restrictions in terms of their extent, reliability or availability, which impede the creation of reliable prediction models [157]. Preclinical research provides vital insights into the mechanisms and effectiveness of drugs by using animal models and in vitro testing. Translating preclinical discoveries to human illness is sometimes difficult because of species disparities, constraints of model systems and insufficient depiction of disease intricacy [158, 159]. The process of developing and verifying predictive models is laborious and requires continual refining and validation using clinical data. Extended periods of development may cause a delay in the use of predictive models for DRP, especially in quickly advancing areas like precision medicine [160].

Biomarkers are essential in drug development since they provide quantifiable indications of disease condition, therapy response or medication toxicity. Nevertheless, the task of developing dependable biomarkers for particular illnesses or medication reactions may be arduous, particularly for intricate or diverse situations [161]. Validating biomarkers requires strong clinical data that establishes their correlation with the advancement of the illness, treatment results or pharmaceutical reactions [162]. This procedure often entails extensive clinical trials and longitudinal investigations, which may be both laborious and expensive. Disease biomarkers and medication response biomarkers might differ across patient groups owing to genetic, environmental or demographic influences. Therefore, it might be difficult to create indicators that can be generally used for DRP activities [162, 163].

The integration of heterogeneous information from genomes, transcriptomics, proteomics, metabolomics and clinical data might improve the creation of prediction models and attempts to uncover biomarkers. Advanced computer techniques, such as ML and artificial intelligence (AI), may be used to analyse extensive biological and clinical data. This analysis aims to detect patterns, correlations and predictive characteristics that are significant for the process of DRP [164]. Cooperation between academics, pharmaceutical corporations, government agencies and nonprofit organisations may promote the exchange of data, the establishment of standardised procedures and joint endeavours to tackle the difficulties of predictive modelling and biomarker identification in DRP [165]. Despite the difficulties, continuous progress in biological research, data science and technology provides hopeful prospects for overcoming the constraints related to predictive modelling and biomarker discovery in DRP. Researchers can expedite the identification and creation of repurposed pharmaceuticals for various therapeutic uses by using multidisciplinary methods and working together [166].

HTS and phenotypic tests allow for the efficient screening of extensive chemical libraries against disease‐relevant biological targets or phenotypic tests. Traditional drug discovery mostly emphasises target‐based strategies, while phenotypic screening may find molecules that have favourable therapeutic effects irrespective of their precise molecular targets [167]. Phenotypic tests, which assess changes in cellular structure, activity or conduct, may provide vital understanding into the pharmacological impacts of repurposed medications. These tests provide a comprehensive viewpoint on drug activity and have the potential to reveal unforeseen therapeutic advantages or mechanisms of action [168]. Progress in stem cell technologies, organoids and samples obtained directly from patients (such as tumour biopsies and patient‐derived xenografts) provides possibilities for developing disease models that closely resemble the physiological conditions [169]. These models may be used for drug screening and the identification of biomarkers. Samples obtained directly from patients may provide valuable information on the diversity of diseases and individual responses to treatments. This can help in the discovery of biomarkers that can predict treatment outcomes and in the development of personalised treatment approaches [170].

### 6.3. Funding and Investment Challenges

Organisations need monetary assets and employees who possess appropriate knowledge in the compound and investigated indications to progress a dropped pharmaceutical candidate. Due to the organisation of drug discovery and development, which is generally centred on certain therapeutic domains, it may be challenging to recognise the potential for repurposing molecules outside of this emphasis within the organisation [171]. Therefore, it is often necessary to have partnerships with several partners in the process of repurposing [172]. Academic researchers may possess the knowledge and skills to analyse compounds, but they may lack the availability of a collection of low‐priority drugs. Similarly, tiny biotechnology firms and academic institutions may need to seek out commercial collaborators in order to overcome budget limitations [173]. In addition, many businesses do not have enough people specifically assigned to the task of outlicencing terminated molecules. As a result, most of these compounds are simply abandoned [174].

Although repurposing holds the potential for being a more cost‐effective and time‐efficient approach compared to developing new compounds from scratch, the process of introducing a recycled substance to the market still incurs significant expenses ranging from several hundred million to billions of dollars, although the initial cost savings result from the elimination of preclinical research [175]. While a significant number of molecules have available data and are well comprehended, repurposing just diminishes, rather than eradicates, the hazards associated with medication development. In the end, repurposing may still need extensive evaluation, and repurposed substances must still go through the same licencing procedure and adhere to the criteria of quality, effectiveness and safety.

The act of repurposing has the potential to save a significant amount of money, potentially amounting to millions of dollars. This is due to its ability to reduce the time spent on preclinical and early‐stage research by 6–7 years, as shown by references [176]. Nevertheless, throughout the advanced phases of clinical development, repurposed drugs might exhibit a failure rate that is comparable to or even greater than that of other compounds, particularly if they have already failed in a primary indication [37]. Even when medications are repurposed, they still need to undergo Phase 2 and 3 clinical trials. These studies exclude 68% and 40% of molecules, respectively, that have made it to that stage, for their new uses [177].

When out‐licencing a substance, there may be significant expenditures associated with remanufacturing the active product and placebo, completing research reports and regulatory paperwork, ensuring drug safety, monitoring patient safety and coordinating activities. Convincing management to dedicate resources to compounds that were previously ineffective is difficult, particularly if the new indication is not a strategic priority [37].

### 6.4. Ethical Considerations and Patient Safety Concerns

DRP, while it holds promise for expediting the development of novel medicines and reducing costs, presents significant ethical difficulties and patient safety concerns. Participants involved in clinical trials for repurposed medications are required to furnish informed consent, demonstrating comprehension of the possible hazards, advantages and uncertainties linked to the experimental therapy [178, 179]. It is crucial to guarantee that individuals have access to unambiguous and thorough information to make well‐informed choices about their involvement. Repurposed pharmaceuticals may possess established safety profiles for their initial applications, but their safety profiles may vary when used for novel purposes or in conjunction with other treatments [180]. Thorough surveillance of patient safety, negative occurrences and interactions between drugs is crucial at every stage of drug development. It is essential to guarantee fair and equal availability of repurposed medications, especially for patients with uncommon or neglected illnesses who may lack access to traditional treatment alternatives [181]. Addressing gaps in medical care availability and medicine prices is a crucial ethical concern in DRP programmes. Pharmaceutical corporations and academics engaged in DRP endeavours are morally obligated to provide pertinent details about the sources, creation and possible constraints of repurposed medications [182]. Clear and open communication builds confidence among parties and facilitates well‐informed decision‐making by patients, healthcare providers and regulatory bodies. The off‐label use of repurposed pharmaceuticals, which refers to the prescription of medications for purposes not officially permitted by regulatory bodies, gives rise to ethical and safety problems. Although off‐label usage may be appropriate in some cases, healthcare practitioners must thoroughly evaluate the existing data, patient preferences and possible hazards before prescribing repurposed medications for off‐label use [183, 184]. Regulatory authorities have a vital role in guaranteeing the safety, effectiveness and quality of repurposed pharmaceuticals by conducting thorough evaluation procedures and monitoring them once they are on the market. Ethical issues include the principles of openness, adherence to regulatory norms and the prioritisation of patient safety throughout the approval and monitoring processes of repurposed pharmaceuticals [185]. The process of making ethical decisions in DRP entails carefully considering the possible advantages of repurposed pharmaceuticals in comparison to the dangers and uncertainties that come with their use. In order to make well‐informed judgements concerning the use of repurposed pharmaceuticals in clinical practice, healthcare professionals, patients and regulatory bodies need to work together to undertake comprehensive benefit‐risk evaluations [186, 187]. Adhering to standards of research integrity, such as honesty, openness and respect for study participants, is essential for ethical conduct in DRP research. Researchers are required to provide precise and truthful accounts of their research results, reveal any potential conflicts of interest and give priority to the well‐being of patients while planning and carrying out repurposing studies [188]. DRP programmes often depend on extensive data gathering and analysis, including patient health records, genetic data and clinical trial data. Safeguarding patient confidentiality and guaranteeing the conscientious use of delicate health data are fundamental ethical considerations [189]. Researchers and organisations are required to comply with stringent data protection requirements and obtain informed permission for the sharing and analysis of data. Continual safety monitoring of repurposed medications is crucial even after they have received regulatory clearance to identify and manage any adverse events, drug interactions and long‐term impacts [190]. Robust means for monitoring the safety of repurposed pharmaceuticals in real‐world clinical practice, such as pharmacovigilance systems, postmarketing surveillance programmes and patient registries, are essential for assuring their ongoing safety [191].

## 7. Future Directions and Emerging Trends

### 7.1. Integration of Multiomics Data for Target Identification

The integration of multiomics data has emerged as a powerful approach in drug development, offering a holistic view of disease mechanisms and therapeutic targets. The specific strategy employed depends on the nature, quality and availability of the omics datasets, as well as the underlying biological hypothesis. A key application of multiomics is the identification and validation of novel drug targets, which may include proteins, genes, metabolites or epigenetic markers involved in disease pathogenesis and progression.

Multiomics techniques can support target discovery in several ways. First, they enable the identification of disease‐specific molecular characteristics by analysing diverse biological layers, such as genomic, transcriptomic, proteomic, metabolomic and epigenomic data, across diseased versus healthy individuals or drug responders versus nonresponders [192]. These techniques allow the detection of variations in DNA methylation, metabolite concentrations, protein expression and gene regulation that correlate with pathological or pharmacological states.

Additionally, integrated omics analysis helps construct molecular networks and signalling pathways that reveal key interactions and mechanistic insights [193]. By mapping associations between genes, proteins, metabolites and epigenetic marks, researchers can better understand disease biology and drug action. Omics‐based prioritization frameworks assess the relevance of candidate targets using criteria such as differential expression, regulatory significance, pathway centrality, functional annotation and disease or drug associations [194].

Once identified, these targets can be experimentally validated using gene knockdowns, overexpression models, site‐specific mutations or pharmacological modulation via inhibitors and activators [195]. Multiomics can also inform the development of computational tools, such as pharmacokinetic/pharmacodynamic (PK/PD) models, systems pharmacology frameworks and ML algorithms—to simulate the therapeutic impact of targeting specific biomolecules [196].

Another key application of multiomics lies in forecasting therapeutic outcomes and optimizing treatment regimens. Drug responses, including efficacy, toxicity, resistance, sensitivity, dosage and duration, can be predicted by analysing biomarkers across omics layers. These insights enable clinicians to tailor drug selection and dosage to individual patients, advancing the goal of PM.

Characterizing inter‐individual heterogeneity in pharmacological responses using multilevel omics data from various biological compounds [197]. Such integrative analysis allows for the classification of patients into subgroups based on drug response phenotypes, for example, responders vs. nonresponders or individuals at high vs. low risk for adverse reactions [198]. Furthermore, multiomics supports the development of predictive models, using ML methods like support vector machines (SVMs), random forests or neural networks, that can estimate various dimensions of drug response, including therapeutic efficacy, safety, toxicity, resistance, sensitivity, dosage and duration [199]. These capabilities advance the goals of PM by guiding tailored treatment strategies based on molecular profiles.

### 7.2. Use of Artificial Intelligence and Machine Learning

The progress of computer AI may greatly diminish the obstacles and constraints of using a computer method for drug repositioning. AI is the replication of human intelligence by equipment, involving abilities such as reasoning, organising, acquiring knowledge and understanding. Its purpose is to optimise the likelihood of attaining various goals, particularly in high‐pressure situations like emergencies or infections with unknown causes. The inevitability of AI’s development is particularly evident in the difficult field of drug discovery and repositioning. The growing abundance of chemical and biological data necessitates the use of advanced computers and algorithms for data mining in order to expedite the drug development process, ensuring rapidness, efficacy and cost‐effectiveness [200].

AI is a component of CADD, which has been used for drug discovery and repositioning for many years. The fundamental principles of ML, like naïve Bayesian classifier (NB), k‐nearest neighbours (KNN) algorithm, logistic regression (LR), Gaussian process and multiple linear regression (MLR) [201], have utilised AI integration to effectively analyse drug repositioning possibilities [202, 203]. Today, the field of ML has progressed to include DL techniques, thanks to advancements in AI. DL approaches enhance data processing capabilities, leading to more trustworthy findings in a shorter time frame and low cost. Ma and colleagues have effectively addressed some problems by using the deep neural network (DNN). Therefore, it is crucial to train and screen a significant number of compounds while also enhancing the accuracy of the quantitative structure–activity relationship (QSAR) approach. This is necessary for accurately predicting both on‐ and off‐target behaviours in drug discovery processes [204]. Medication–target interaction discovery is a crucial issue to consider in DRP. The drug repositioning process relies on conventional ligand and structure‐based methods, which use QSAR to forecast the biological functions of the targeted molecules. This approach assumes that structurally similar molecules will have identical biological operations and uses genuine molecular docking simulations [200]. Nevertheless, the constraints of these conventional computing techniques highlight the significance of DL approaches. An example of a DL‐based algorithm framework, called DeepDTIs, has effectively and correctly predicted novel drug–target interactions (DTIs) between licenced medications and targets. This prediction is achieved despite the need to categorise the targets into distinct groups. Studies have shown that the DeepDTIs algorithm surpasses other ML approaches, including the random forest [205] and SVM. This technique has significant promise for predicting novel pharmacological targets based on current targets or predicting the relationship of new targets with existing medications [206].

During critical periods like as the ongoing COVID‐19 epidemic, AI has played a significant role in supporting drug repositioning efforts. An example of this is BenevolentAI, an AI prediction method that utilises a comprehensive collection of organised medical data. This database contains numerous connections extracted from scientific literature using ML. BenevolentAI has been instrumental in identifying potential drug repositioning candidates for the treatment of COVID‐19. The investigation has shown that the ACE‐2 cell receptor, which is present in large quantities in the blood vessels, heart and lung AT2 alveolar epithelial cells, plays a crucial role in the infection of SARS‐CoV‐2, resulting in COVID‐19 [207]. AAK1, a protein kinase linked with AP2, controls the ACE‐2 cell receptor, which is a vital receptor for blocking the entrance of SARS‐CoV‐2. Baricitinib, a Janus kinase inhibitor, was identified using this method. It has been shown to have excellent therapeutic results at a dosage of 2–4 mg once daily. It inhibits the AAK1 enzyme, which contributes to its effectiveness in treating the condition while minimising negative side effects [208]. Overall, the combination of CTs and AI has significant potential for medication repositioning, particularly in the context of the COVID‐19 pandemic, where there is a pressing need for effective treatments. Furthermore, in some instances, this methodology exhibits similar or superior performance compared to in vitro experiments.

### 7.3. Personalized Medicine Approaches in Drug Repurposing

PM methods in DRP include customising therapy regimens for individual patients according to their distinct genetic, molecular and clinical attributes [209]. Employ high‐throughput molecular profiling tools, like as metabolomics, genomics, proteomics and transcriptomics, to analyse the molecular makeup of illnesses and discover biomarkers unique to individual patients [210]. Utilise omics data analysis to categorise patients into distinct categories according to their molecular profiles, facilitating the selection of individuals who may react positively to repurposed medications. Find patient subgroups based on molecular factors that are linked to the effectiveness or resistance of repurposed medications [211]. Determe genetic variations, gene regulation patterns or pathway stimulation that may be used to anticipate the effectiveness of certain repurposed medications. Exploring the use of combo medicines by repurposing pharmaceuticals that target distinct routes or mechanisms of action [212]. Constructing personalised combination therapies for specific patients by considering their molecular profiles and illness features to improve effectiveness and reduce adverse effects. Utilise pharmacogenomic data to anticipate individual variances in medication response and toxicity [213]. Determe genetic variations that impact medication metabolism, transport or pharmacodynamics to enhance drug selection and dosage in personalised treatment plans. Develop a system to continuously track the effectiveness of therapy and the advancement of the illness, utilising wearable devices, biomarker tests or imaging technology [214]. Optimise therapy results by adapting treatment tactics according to the dynamic fluctuations in patient biomarkers and clinical parameters. Create clinical decision support systems that include patient‐specific molecular data, clinical information and evidence‐based recommendations [215]. Offer doctors tailored treatment suggestions and decision support tools to assist with DRP techniques and enhance patient outcomes. Encourage individuals to actively engage in treatment choices and personalised medicine efforts [216]. Inform patients on the significance of molecular profiling and personalised treatment methods in DRP, promoting well‐informed decision‐making and adherence to therapy. Resolve the regulatory obstacles linked to personalised medicine strategies in DRP, which include verifying biomarkers, designing clinical trials and establishing approval routes [217]. Engage in cooperation with regulatory bodies to develop frameworks for assessing and authorising personalised DRP methods. By incorporating the ideas of personalised medicine into DRP initiatives, scientists and medical professionals may enhance treatment results, reduce negative side effects and enhance patient care across different illness contexts [218]. Customised methods show potential for improving the effectiveness and accuracy of DRP procedures, eventually resulting in more efficient treatments for particular patients [219].

### 7.4. Strategic Opportunities for the Future

The future of DRP lies in its integration into broader precision medicine and open‐science frameworks. Strategic opportunities include global databases that track off‐label uses and post‐market safety signals, public–private partnerships that facilitate compound sharing, and adaptive trial designs that validate repurposed drugs more efficiently. Regulatory bodies such as the FDA and EMA are increasingly open to innovative approval pathways like the 505(b)(2) mechanism, which streamlines the approval of repurposed agents [220]. Moreover, the use of real‐world evidence (RWE) and longitudinal patient registries can complement traditional clinical trials, offering new avenues for validation. Building multidisciplinary collaborations across academia, regulatory agencies and industry is key to unlocking the full potential of this approach. Investing in DRP not only bridges the translational gap but also ensures equitable access to life‐saving therapies globally.

## 8. Conclusion

Drug repurposing offers a powerful, efficient pathway for translating existing pharmacological knowledge into new therapeutic solutions. While numerous successes have demonstrated its clinical and economic value, challenges such as regulatory ambiguity, IP limitations and lack of predictive biomarkers persist. Looking ahead, an integrated framework combining computational modelling, RWE and collaborative clinical trials can accelerate DRP pipelines. Public–private partnerships, harmonized regulatory pathways and adaptive trial designs will play a pivotal role. Ultimately, embedding DRP within a PM framework, guided by genomics, pharmacovigilance data and AI‐driven drug‐matching algorithms, can transform treatment paradigms and expand access to targeted therapies worldwide.

**Nomenclature**
DRP: Drug repurposing
R&D: Research and development
HTS: High‐throughput screening
CTs: Computational techniques
EHR: Electronic health records
ENL: Erythema nodosum leprosum
MM: Multiple myeloma
CVDs: Cardiovascular diseases
NDs: Neurodegenerative disorders
PD: Parkinson’s disease
DARA: Direct‐acting repurposed antivirals
FDA: Food and Drug Administration
HTRA: Host‐targeting repurposed antivirals
ML: Machine learning
AD: Alzheimer’s disease
CADD: Computer‐aided drug design
GO: Gene Ontology
KEGG: Kyoto Encyclopedia of Genes and Genomes
PPI: Protein‐protein interaction
CRC: Colorectal cancer
ACCD: Aberrant Cell Cycle Diseases
iPSCs: Induced Pluripotent Stem Cells
PM: Personalized medicine
HCV: Hepatitis C virus
autoAbs: Autoantibodies
LNA: Locked nucleic acid
Abs: Antibodies
HIV: Human immunodeficiency virus
CMV: Cytomegalovirus
MS: Multiple sclerosis
HSV: Herpes simplex virus
RA: Rheumatoid arthritis
SSRIs: Selective serotonin reuptake inhibitors
SNRIs: Serotonin–norepinephrine reuptake inhibitors
HF: Heart failure
CRP: C‐reactive protein
IP: Intellectual property
IPR: IP rights
EMA: European Medicines Agency’s
FTO: Freedom to operate
AI: Artificial intelligence
CNVs: Copy number variations
SNPs: Single nucleotide polymorphisms
NB: naïve Bayesian classifier
KNN: k‐nearest neighbours
LR: Logistic regression
MLR: Multiple linear regression
DNN: Deep neural network
QSAR: Quantitative structure‐activity relationship
DTIs: Drug–target interactions
SVM: Support vector machine.

## Ethics Statement

The authors have nothing to report.

## Consent

The authors have nothing to report.

## Conflicts of Interest

The authors declare no conflicts of interest.

## Author Contributions

Conceptualization, Mohamed El‐Tanani, Reyaz Hassan Mir, Md Sadique Hussain, Prince Ahad Mir and Adil Farooq Wali; methodology, Javedh Shareef, Roohi Mohi‐ud‐din and Sirajunisa Talath; formal analysis, Sathvik B. Sridhar; Javedh Shareef; Nishant Kumar and Adil Farooq Wali; investigation, Mohamed El‐Tanani, Reyaz Hassan Mir, Manjunatha Goud, Imran Rangraze Nishant Kumar and Adil Farooq Wali; writing—original draft preparation, Reyaz Hassan Mir, Md Sadique Hussain, Manjunatha Goud, Imran Rangraze Nishant Kumar and Adil Farooq Wali; and Sathvik B. Sridhar; writing—review and editing, Sirajunisa Talath; Prince Ahad Mir;. Javedh Shareef, Roohi Mohi‐ud‐din and Adil Farooq Wali. All authors have read and agreed to the published version of the manuscript.

## Funding

No funding was received for this manuscript.

## Data Availability

The data that support the findings of this study are available from the corresponding author upon reasonable request.
